# Genetically manipulated phages with improved pH resistance for oral administration in veterinary medicine

**DOI:** 10.1038/srep39235

**Published:** 2016-12-15

**Authors:** Franklin L. Nobrega, Ana Rita Costa, José F. Santos, Melvin F. Siliakus, Jan W. M. van Lent, Servé W. M. Kengen, Joana Azeredo, Leon D. Kluskens

**Affiliations:** 1 CEB – Centre of Biological Engineering, University of Minho, Campus de Gualtar, 4710-057 Braga, Portugal; 2 Laboratory of Microbiology, Wageningen University and Research Centre, Stippeneng 4, 6708 WE Wageningen, The Netherlands; 3 Laboratory of Virology, Department of Plant Sciences, Wageningen University, Droevendaalsesteeg 1, 6708 PB Wageningen, The Netherlands

## Abstract

Orally administered phages to control zoonotic pathogens face important challenges, mainly related to the hostile conditions found in the gastrointestinal tract (GIT). These include temperature, salinity and primarily pH, which is exceptionally low in certain compartments. Phage survival under these conditions can be jeopardized and undermine treatment. Strategies like encapsulation have been attempted with relative success, but are typically complex and require several optimization steps. Here we report a simple and efficient alternative, consisting in the genetic engineering of phages to display lipids on their surfaces. *Escherichia coli* phage T7 was used as a model and the *E. coli* PhoE signal peptide was genetically fused to its major capsid protein (10 A), enabling phospholipid attachment to the phage capsid. The presence of phospholipids on the mutant phages was confirmed by High Performance Thin Layer Chromatography, Dynamic Light Scattering and phospholipase assays. The stability of phages was analysed in simulated GIT conditions, demonstrating improved stability of the mutant phages with survival rates 10^2^–10^7^ pfu.mL^−1^ higher than wild-type phages. Our work demonstrates that phage engineering can be a good strategy to improve phage tolerance to GIT conditions, having promising application for oral administration in veterinary medicine.

Bacteriophages or phages are considered one of the most promising alternatives to antibiotics due to their natural antimicrobial properties. These bacteria-infecting viruses of high-specificity have applications on a wide range of fields including agriculture, food, and human or animal therapy^1^.

In the veterinary field, phage therapy has been evaluated against several zoonotic pathogens, with the intent to control transmission of diseases to humans and to reduce economic loss^2^,^3^,^4^,^5^. Some successful applications have been reported, including treatment of infections of *Escherichia coli* in calves, pigs, lambs and poultry^6^,^7^,^8^,^9^, of *Campylobacter* in poultry^10^,^11^, of *Salmonella* in poultry and pigs^12^,^13^, *Clostridium perfringens* in poultry^14^ and *Pseudomonas aeruginosa* in dogs^15^. Currently, some phage products are already being commercialized (such as BioTector^®^S1 and INT-401^TM^ to control *Salmonella* in poultry feed and *Clostridium perfringens* infections, respectively)^14^.

Despite some successful attempts, phage therapy in veterinary has presented variable results. These disparities are in part due to the severe physiochemical conditions that phages encounter *en route* through the digestive system and at the site of infection^16^,^17^. Indeed, phage viability and survival have been shown to be affected by factors as acidity and temperature, which cause modifications on phage’s structural components and nucleic acids^18^. Acidity of the gastric environment of most animals is a major concern for oral administration of phages, since the low pH values significantly reduce phage titre and proliferation^19^,^20^,^21^,^22^. The body temperature of animals (e.g. 38 °C in dogs and 42 °C in chickens) is also a parameter that can affect treatment efficacy since temperatures above the optimum prolong the phage’s latent period, and below the optimum reduce phage penetration and consequent proliferation. Enzymes found in the gastrointestinal tract (GIT), such as pepsin in gastric fluid, and amylase, lipase and protease in pancreatic fluid, may also cause degradation of phages^23^,^24^,^25^,^26^.

Because of the challenges faced by orally-delivered phages, it is desirable to provide additional protection to enhance their survival in the GIT. Phage encapsulation in natural biopolymer-derived matrices have been used as a strategy to improve phage stability^27^,^28^,^29^,^30^,^31^. An increased survival of phages has been obtained with this technology in the GIT of cattle, pigs and poultry^27^,^32^,^33^. However, for this type of strategy, the selection of the biopolymer is critical, and some important features have to be met, such as the ability to be synthesized under mild environmental conditions, and to be easily tailored, non-toxic and environmental friendly (e.g. not requiring the use of organic solvents^34^)^29^,^35^.

Here we suggest an alternative to encapsulation, based on the display of phospholipids on the surface of the phages by genetic engineering. Such a “natural coating” may provide the phage with added protection against the acidic environment and other hostile conditions found in the GIT of animals, potentially without affecting the phage’s infection ability. The genetic engineering of lytic phages has proven difficult in the past, but with the development of new techniques such as Bacteriophage Recombineering of Electroporated DNA (BRED)^36^,^37^ it is now becoming a simple process, cheaper than encapsulation and without special requirements for equipment or reagents.

In this work we used the well-known *E. coli* phage T7 as a model, and modified its genome with the *E. coli* outer membrane phosphoporin protein E (PhoE) signal peptide (sequence MKKSTLALVVMGIVASASVQA). Signal peptides are temporary N-terminal extensions of proteins that are destined to be exported from the cytoplasm of bacteria^38^,^39^. These peptides have little sequence homology but they share certain properties such as a positively charged N-terminal region, a central hydrophobic core, and a more polar C-terminal region containing the cleavage site^40^,^41^. Signal peptides in *E. coli*, as the PhoE signal peptide, are thought to be directly involved in the binding of proteins to the membrane by electrostatic interactions between the N-terminus and anionic membrane phospholipids, thereby initiating membrane translocation of the protein^42^. Biophysical studies have also demonstrated a strong interaction between isolated signal peptides and lipids of model membrane systems^43^,^44^,^45^. Here we intend to explore the properties of the PhoE signal peptide by fusing it to the major capsid protein (10 A) of phage T7. We hypothesize that the presence of the PhoE signal peptide on the capsid of the phage may provide added protection to the phage by the interaction of the signal with *E. coli* phospholipids. These lipids, which are synthesized at the cytoplasmic side of the inner membrane^46^, may be acquired during phage replication inside the bacteria, thereby creating a lipidic coating (Supplementary Fig. S1). Indeed, the attachment of phospholipids to the major capsid protein was confirmed by surface analysis using High Performance Thin Layer Chromatography (HPTLC), Dynamic Light Scattering (DLS) and phospholipase assays. Moreover, these mutant phages demonstrated a higher stability than the wild-type phages under different conditions simulating those found in animal’s GIT (pH, temperature and enzymes). Altogether, we show that lipid coating of phages obtained by genetic manipulation represents a promising alternative to encapsulation for orally delivered phage therapy.

## Results and Discussion

Oftentimes, acid-lability contributes to a poor efficacy of phage therapy on the GIT of animals. In this study we therefore focused on the development of a solution to overcome acid lability of orally administered phages. For that we fused the *E. coli* PhoE signal peptide to the major capsid protein (gp10A), using *E. coli* phage T7 as a model phage. Using BRED we inserted the PhoE signal peptide into the T7 genome, fused to the major capsid protein (gp10A), obtaining the mutant phage T7::PhoE. When transcribed, the assembled mutant phage may bind phospholipids from the inner leaflet of the cytoplasmic membrane upon ionic interaction with the polar head group of e.g. phosphatidylglycerol (main anionic phospholipid in *E. coli* and hence expected to be the phospholipid attached to the mutant phages).

### Assessment of phage mutation, stability and infectivity

After BRED the insertion of the PhoE signal peptide into the T7 phage genome was confirmed by PCR (Supplementary Fig. S2), and by sequencing we could show that the mutation remained stable for at least 10 generations.

Since the generated mutations may have affected the infection ability of the phages, the infectivity of the mutant phage T7::PhoE was evaluated and compared to the wild-type T7. The infection parameters were assessed through a one-step growth curve. For both phages the latent period was established at approximately 15 min, with an eclipse time around 10 min, and a burst size of 253 ± 30 for T7 and 243 ± 92 for T7::PhoE. Apparently, the mutant phages showed an infectivity identical to the wild type phage, indicating no effect of the mutation on phage replication. Further results obtained are therefore not a consequence of any alteration on the capacity of the phage to infect.

### Confirmation of phospholipids on the mutant phage capsid

The wild-type T7 and mutant T7::PhoE phages were inspected by transmission electron microscopy (TEM) in an attempt to visualize the presence of a coating surface around the mutant phages. However, no clear differences could be seen by TEM (see Supplementary Fig. S3). Osmium tetroxide staining was also attempted due to its capacity to stain lipids; however, again, no differences could be seen (Supplementary Fig. S3). So other strategies were followed to check for the presence of lipids on the mutant phages, namely surface analysis by HPTLC, DLS and a phospholipase assay.

**Figure 1:**
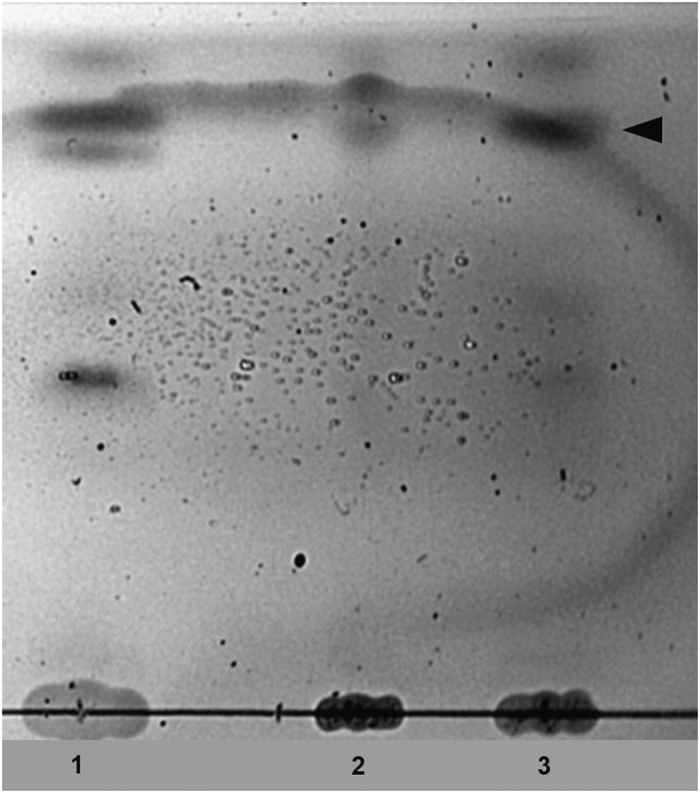
HPTLC chromatogram of total lipid extracts from phage isolates. Lane 1: *Escherichia coli* BL21 positive control, lane 2: wild-type phage T7; lane 3: mutant phage T7::PhoE. Arrow head indicates the lipid species that is particularly enriched in the T7::PhoE mutant.

For HPTLC, the total lipids were extracted from wild-type T7 and T7::PhoE phages, as well as from the host bacteria, and analysed on HPTLC plates. The separated lipids were visualized by sulfuric acid charring. The chromatogram (Fig. 1) shows an increased lipid content of the mutant phages (Fig. 1, lane 3) compared to the wild-type control (Fig. 1, lane 2).

Here we observed an increased intensity of one or several lipids/phospholipids species in the mutant, which is comparable to the *E. coli* total lipid profile (Fig. 1, lane 1). Of interest is the fact that one specific lipid species increased its intensity in particular (arrow head in Fig. 1), as the signal peptide is known to bind only anionic lipids. This data supports the incorporation of *E. coli* lipids/phospholipids onto the capsid structure of the mutant phage.

**Figure 2:**
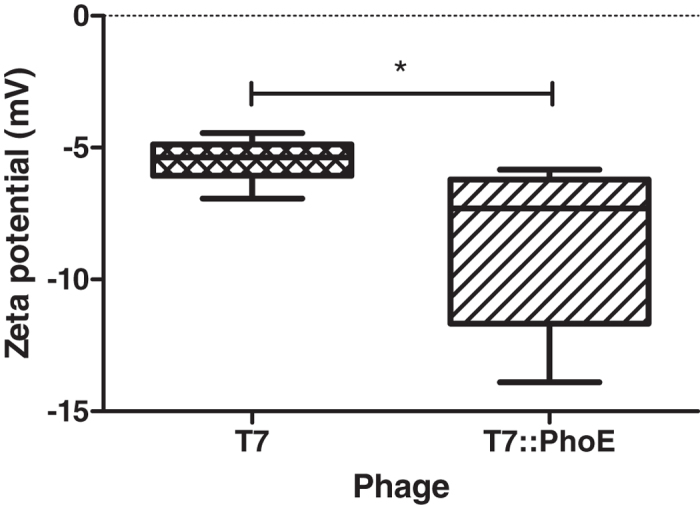
Zeta potential of phages T7 (wild-type) and T7::PhoE (mutant) determined by DLS. Statistical differences (*P* < 0.05) obtained using t-test analysis are represented by *.

Additionally, the zeta potential of the wild-type and mutant phages was determined by DLS (Fig. 2). The charge of the phage particles was significantly different (*P* = 0.0154), with average values of −5.49 mV and −8.69 mV, for T7 and T7::PhoE respectively. Since the PhoE signal peptide has a predicted positive net charge of +2 mV (http://pepcalc.com/protein-calculator.php), these results indicate that another factor is reducing the mutant phage’s overall surface charge, which may well be a consequence of attached lipids/phospholipids, recruited by the PhoE signal peptide. For instance, the most prevalent anionic phospholipid in *E. coli,* phosphatidylglycerol, present at the cytoplasmic side of the inner membrane, has been reported to have a negative charge at the phosphate moiety of the head groups, which may be responsible for the increased negative charge found by DLS for the mutant phages.

### Phage survival at different pH values

**Figure 3:**
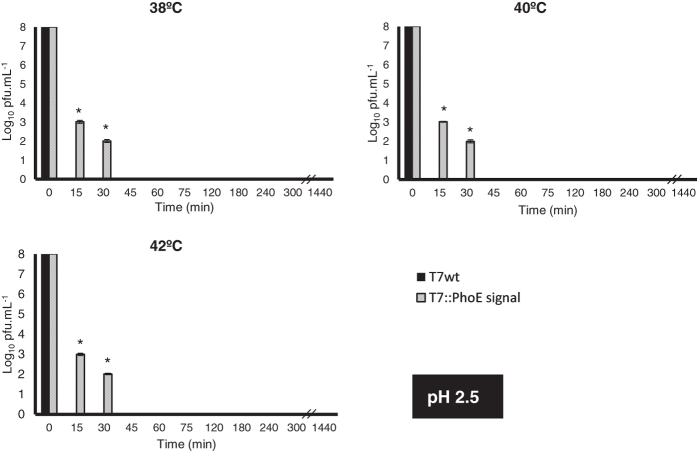
Survival of wild-type T7 and mutant T7::PhoE phages to different conditions that simulate those found in the GIT of animals: pH of 2.5, temperature range from 38 °C to 42 °C. Statistical differences (*P* < 0.05) obtained using t-test analysis are represented by *.

**Figure 4:**
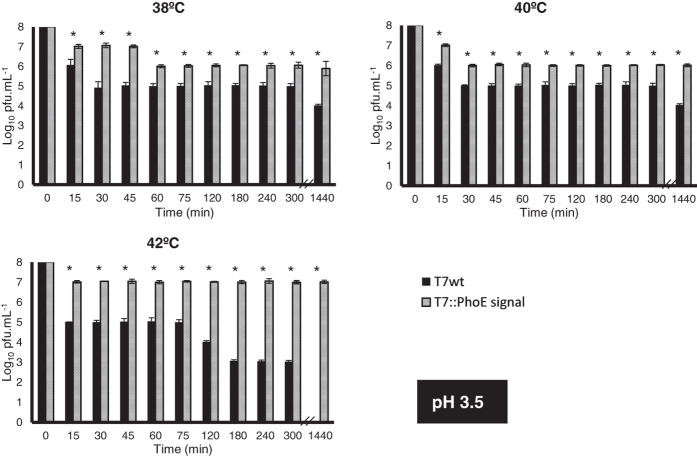
Survival of wild-type T7 and mutant T7::PhoE phages to different conditions that simulate those found in the GIT of animals: pH of 3.5, temperature range from 38 °C to 42 °C. Statistical differences (*P* < 0.05) obtained using t-test analysis are represented by *.

**Figure 5:**
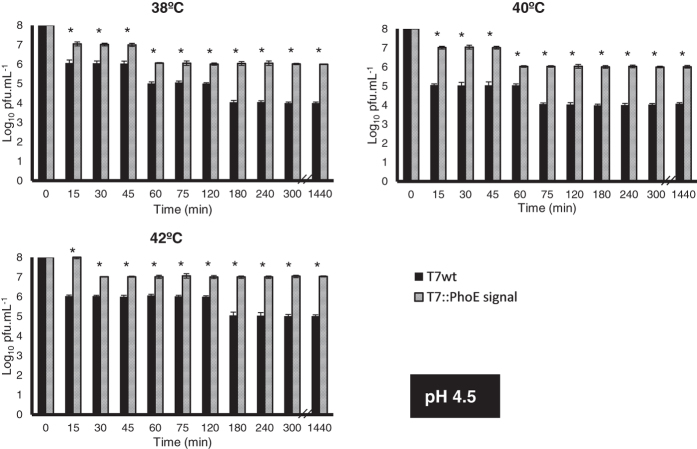
Survival of wild-type T7 and mutant T7::PhoE phages to different conditions that simulate those found in the GIT of animals: pH of 4.5, temperature range from 38 °C to 42 °C. Statistical differences (*P* < 0.05) obtained using t-test analysis are represented by *.

**Figure 6:**
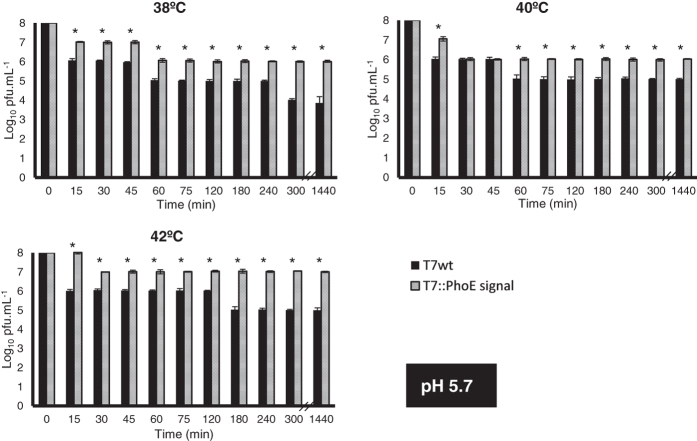
Survival of wild-type T7 and mutant T7::PhoE phages to different conditions that simulate those found in the GIT of animals: pH of 5.7, temperature range from 38 °C to 42 °C. Statistical differences (*P* < 0.05) obtained using t-test analysis are represented by *.

Phages can become irreversibly damaged by exposure to low pH values (as well as other conditions, see review^17^), a condition typically found in the GIT of animals. The display of a coating on the surface of phages may provide stability, protecting them from these conditions. To evaluate this, the wild-type and mutant phages were used in *in vitro* survival assays simulating different conditions encountered along the GIT of animals. The conditions tested included: i) temperature, ranging from 38 °C to 42 °C to represent typical body temperatures of different animals; ii) pH, ranging from 2.5 to 5.7 to simulate the acidic environment found in different compartments of the GIT; and iii) time, ranging from 15 to 1440 min (24 h) to represent different residence times of each compartment of the GIT. Results can be seen in Figs 3, 4, 5 and 6. Under the most severe condition, at a pH value of 2.5 (Fig. 3), found for example in the gizzard of poultry, the wild-type phage’s titre is completely lost in just 15 min, while the mutant phages can survive up to 30 min with a 10^6^-fold decrease in titre. For pH values 3.5 (Fig. 4), 4.5 (Fig. 5) and 5.7 (Fig. 6), the mutant phage’s titre is generally reduced by a factor of 10^1^ or 10^2^, whereas the wild-type phage’s titre generally reduces by a factor of 10^2^ to 10^4^. This average 100-fold difference between mutants and wild-type phages can have a significant impact on the course of oral phage therapy. In fact, it is reported that, for oral application of phages, doses as high as 10^11^–10^13^ pfus.mL^−1^ are required to ensure that a significant number of phages (at least 10^6^) reaches the intestines^46^. So, the difference observed here for phage survival may potentially result in a higher amount of phages reaching the target site and consequently improves treatment efficacy (Fig. 3, 4, 5 and 6).

It is possible that the presence of the lipid/phospholipid coating the surface of the mutant phages limits the direct contact of the phage with the acidic environment, therefore resulting in the improved viability. Our results are comparable to those obtained by Dini *et al*.^27^ for phage CA933P, Ma *et al*.^32^ for phage Felix O1, and Samtlebe *et al*.^31^ for phage P008, using encapsulation methods. Both observed increased survival of encapsulated phages at acidic pH values when compared to free phages.

Also interesting to note are the strong differences between the mutant and wild-type phages observed for the higher temperatures and longer incubation times. This is particularly accentuated for pH 3.5 (Fig. 4), at temperatures of 42 °C, where a 10^4^ to 10^7^ fold difference can be observed after 180 min and 1440 min of incubation, respectively. So it is possible that additionally to pH, the lipid coating provides protection against increased temperatures, which may affect the activity of temperature-sensitive phages. This may be an important characteristic to be considered in therapeutic applications, especially due to the range of body temperatures observed in different animals, making the mutant phages more broadly applicable, and tolerant to the high temperatures (fever) of the body when fighting an infection.

An important aspect to consider is that the lipid/phospholipid coating generated by our mutation only provides protection to the capsid of the phage. The tail and the fibres, which are the phage components responsible for recognition and infection in tailed phages^47^, are non-modified and therefore remain exposed to the acidic pH values. This suggests that damage caused by low pH values on phages is not necessarily a consequence of an effect on the phage’s proteinaceous structure, otherwise the tails would have been degraded and the phage would not infect. The damage may instead be elicited on the genetic material of the phage, with the phospholipid coating on the capsid preventing this effect. On the other hand, it is known that phages aggregate at acidic pH due to a protonation effect, adversely impacting their activity; with an increased negative charge in the mutant phages, it is possible that protonation is not sufficient to cause complete aggregation of the phages, thereby having a lower impact on their activity. To prove that phages retain their capacity to infect after being subjected to low pH values, we tested whether the mutant phages retained their capacity to multiply in the bacteria after being subjected to the acidic environment. After 3 h of incubation with the bacterial host, the phages’ titre increased 100-fold (data not shown). This indicates that the phages are still able to infect and multiply after being subjected to the acidic conditions that may be encountered in the stomach, a promising result for the treatment of bacterial infections in the gut. So, the detrimental effect of an acidic pH must involve other factors, and should be explored in future studies.

### Mutant phage sensitivity to phospholipases

**Figure 7:**
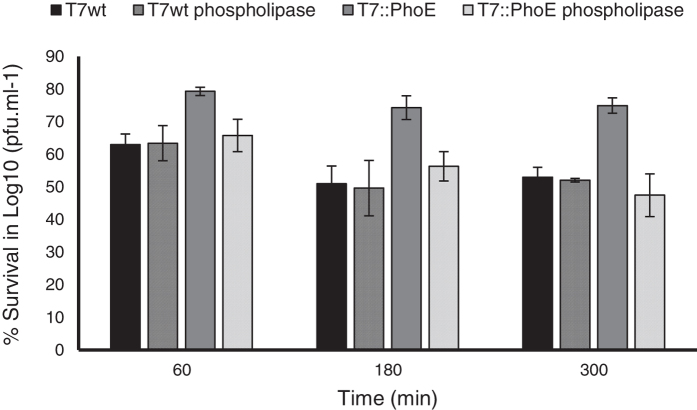
Survival of wild-type T7 and mutant T7::PhoE phages to a pH of 4 when subjected and not subjected to the activity of phospholipase A2. Statistical differences (*P* < 0.05) obtained using t-test analysis are represented by *.

The pH-resistant phenotype of the mutant phage demonstrated above led us to further evaluate the hypothesis of it being caused by the presence of phospholipids attached to the PhoE signal on the capsid of the mutant. For this, reverting assays using enzymes able to specifically degrade phospholipids, i.e. phospholipases, were performed. In these assays, both wild-type and mutant phages were subjected to the activity of phospholipase A2. This enzyme recognizes and hydrolyses the sn-2 acyl bond of phospholipids, forming a fatty acid and a lysophospholipid. Figure 7 demonstrates that after phospholipase activity, the mutant phage presents a log survival similar to the wild-type phage (both subjected and non-subjected to the activity of phospholipase), and lower than the control mutant phage (not subjected to phospholipase). The reversion of the mutant phage to a pH-sensitive phenotype caused by the phospholipase further confirms the presence of phospholipids on the capsid of the mutant, and indicates their involvement on tolerance to low pH.

It must be pointed out that phospholipase A2 is present in the pancreatic fluids secreted into the gut^48^, and may thus revert the protected effect of phospholipids on phage capsids of the mutant phages. However, once in the gut, phages will no longer be subjected to low pH values and therefore do not require the additional protection provided by phospholipids. Thus it is expected than even if phospholipase A2 acts on T7::PhoE, the therapeutic effect of the phage will not be compromised.

### Phage stability in enzymatic fluids and bile salts

**Figure 8:**
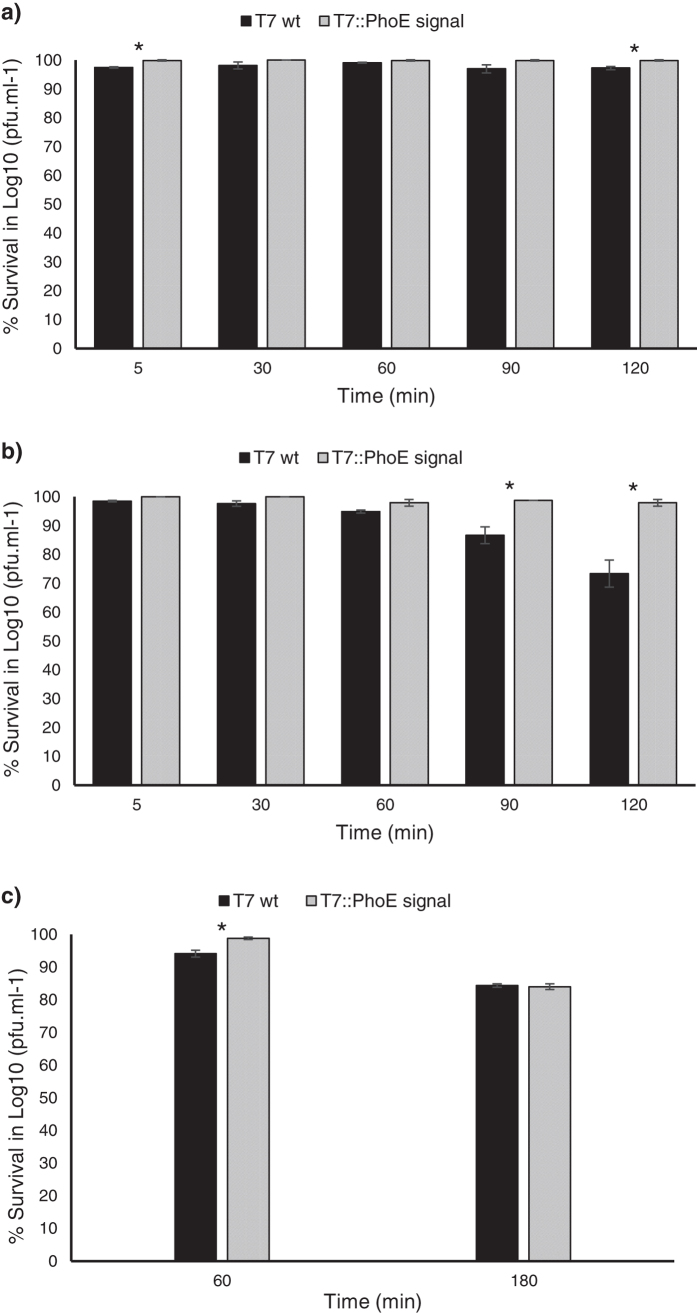
Stability of wild-type T7 and mutant T7::PhoE phages under simulated conditions. (**a**) pancreatic fluid, (**b**) gastric fluid, (**c**) bile fluid. Statistical differences (*P* < 0.05) obtained using t-test analysis are represented by *.

Phages administered orally must withstand the passage through the gastric fluid to be of therapeutic use. This does not only mean survival at low pH values but also resistance to hydrolytic enzymes as pepsin (stomach), lipase, amylase and protease (pancreas). Therefore, the stability of the wild-type and mutant phages under simulated gastric and pancreatic conditions was evaluated (Fig. 8a and b). Exposure to pancreatic enzymes (Fig. 8a) at concentrations within the range found in duodenal juice (around 1.4 ± 0.7 mg/mL^49^) had no major effect on the viability of either wild-type or mutant phages, although significant differences (*P* < 0.05) can be found between phages after 120 min of incubation. But in general pancreatic enzymes do not appear to be a major concern for the oral delivery of phage T7, with less than 5% reduction in the phage titre.

On the other hand, gastric enzymes (Fig. 8b) lead to a substantial reduction in the number of wild-type T7 at 90 min and 120 min, while the mutant T7::PhoE remained mostly unaffected (*P* < 0.05). As already discussed for pH survival, the presence of lipids/phospholipids on the surface of the mutant phages may limit the access of the enzymes to the phage and thereby prevent degradation. The protection provided by the lipid coating on the mutant phages appears to be similar^27^ or even superior^32^ to that observed with microencapsulation approaches, where the microencapsulated phages had a comparably higher rate of survival than free phages in simulated gastric fluid, but still with partial loss of viability (2.58 log units after 1 h).

Bile acid is secreted into the GIT, where it works as an anionic surfactant. Some phages have already demonstrated resistance to the effect of bile^50^ but others appear to be somewhat affected^32^. So in this assay the stability of the wild-type T7 and mutant T7::PhoE in simulated bile fluid was determined (Fig. 8c). Bile salts affect the wild-type phage significantly more (*P* < 0.05) than the mutant phage after an incubation of 1 h, but shows similar effects after 3 h, with a viability reduction of about 16% (corresponding to 1 log). However, it should be emphasized that we used a bile concentration (2%) which exceeds to that found in bile produced in the organism (around 0.7%^51^), so the effect of bile salts on these phages is expected to be lower than those observed here.

In summary, in this study we demonstrate that an engineered phospholipid coating of the T7 phage acts as a protective barrier, significantly improving phage survival under conditions simulating those found on the GIT of different animals, in particular acidity and hydrolytic enzyme activity. Compared to other methods to date, such as microencapsulation, our approach has the advantages of process simplicity, with significantly less optimization steps and a simple process for scale-up (requiring only phage amplification), being equally efficient but with improved viability/stability.

Importantly, we show that phage engineering may be a feasible, simple and cost-effective approach to improve phage properties for oral administration in animals. Furthermore, the insertion of the PhoE signal on the capsid of phage T7 allows future exploitation of the frameshifting on gene 10, characterized by the expression of two products, the major 10 A protein, and the minor 1α protein, which results in different phage phenotypes^52^. So, replication of the mutant T7::PhoE in other hosts (different *E.coli* strains) could change the copy number of 10 A resulting in different phenotypes of the same mutant. We hypothesize that such mechanism could be explored to relax or tighten the coating effect for other therapeutic applications and/or other administration routes. Other phages with this dynamic frameshifting, e.g T7-like phages. T3-like and T4-like phages can also be explored to display similar coatings for other applications.

Another relevant aspect not studied here is the possibility to increase phage-host encounters by sub-diffusive motion in mucosal surfaces^53^. The mucus rich environment in the GIT has been proven to be the principal location of most translocation events of bacterial pathogens. Future *in vivo* studies can test the ability of our mutant phage to survive for a longer period in the gut, by fast absorption to and diffusion into the mucosa, which increases the encounters with the pathogenic host and may improve the efficacy of phage therapy.

## Methods

### Buffers and media

Saline-Magnesium (SM) buffer, containing 100 mM NaCl, 8 mM MgSO_4_.7H_2_O and 50 mM Tris (pH 7.5) was used for phage dilutions. Lysogeny broth (LB) medium^54^ was used for bacterial growth. Super Optimal broth with Catabolite repression (SOC) was used to recover cells after recombineering. Agar was used at a concentration of 1.2% in plates and 0.7% in soft agar. Ampicillin (Amp) was used at a final concentration of 100 μg.mL^−1^. L-arabinose at 10% (w/v in water) was used to induce recombineering functions.

### Bacteriophages, bacteria and plasmids

Phage T7 was kindly provided by Prof. Ian J. Molineux, University of Texas. The phage host was *E. coli* BL21 (Stratagene). Plasmid pKD46, an ampicillin-resistant and temperature sensitive plasmid that encodes the lambda Red genes (*exo, beta* and *gam*) was used to prepare recombineering competent cells.

### Preparation of recombineering competent *E. coli* BL21 cells

Electrocompetent *E. coli* BL21 cells were prepared as described previously, with minor modifications^55^. Briefly, cells were grown in LB medium to mid-log phase and harvested by centrifugation (3000 × *g*, 4 °C, 15 min). The culture was concentrated 500-fold by washing three times with ice-cold sterile 10% (v/v) glycerol, and suspended in a final volume of 80–300 μL. Then, 100–500 ng of pKD46 were electroporated into aliquots of 20 μL electrocompetent cells at 1.8 kV and 25 pF using a Gene Pulser XCell Microbial System (BioRad). Cells were recovered with SOC and incubated for 1–2 h at 37 °C, 200 rpm. Cells were spread in LB plates containing ampicillin (LB-Amp) and incubated overnight at 30 °C. Transformed *E. coli* BL21::pKD46 colonies were picked and grown in fresh LB-Amp medium for a few hours, followed by plasmid extraction (NucleoSpin^®^ Plasmid (NoLid) kit, Macherey-Nagel) and digestion with the restriction enzyme BamHI-HF (New England Biolabs) to confirm the presence of pKD46.

### Construction of the recombineering substrates

For the construction of the recombineering substrates, the primers (Life Technologies, Portugal) presented in Supplementary Table S1 were used at a concentration of 25 μM for Knight - annealing and primer extension with Taq polymerase. The forward and reverse primers of the PhoE signal peptide were mixed with water, 10x buffer Taq A, dNTPs and Kapa Taq DNA polymerase (Kapa Biosystems), and annealed and extended using the following program: 95 °C for 5 min; 5 cycles of 95 °C for 30 s, 50 °C for 30 s, and 72 °C for 30 s; and 72 °C for 5 min. The resultant constructs were purified using the GRS PCR & Gel Band Purification Kit (Grisp) and sizes confirmed on a 1% SGTB agarose (Grisp) gel using the Low Molecular Weight DNA ladder (New England Biolabs).

### Bacteriophage Recombineering of Electroporated DNA

Engineering of the phage T7 genome was based on the method described by Marinelli *et al*.^36^,^37^ with some modifications. Briefly, *E. coli* BL21::pKD46 was grown to early-log phase at 30 °C in 100 mL LB-Amp medium, and the expression of lambda Red by pKD46 was induced for 30 min with L-arabinose (0.1% final concentration). Cells were then infected with T7 at a multiplicity of infection (MOI) of 1–3 for the eclipse time (10 min). Cells were then made electrocompetent as described above. 100–500 ng of the substrate were electroporated into the electrocompetent cells at 1.8 kV and 25 pF, and cells were recovered with SOC medium for 1–2 h at 37 °C, 200 rpm. The suspension was then mixed with approximately 3 mL soft agar and 100 μL of an *E. coli* BL21 culture, poured onto LB plates, and incubated overnight at 37 °C.

### Recovery and confirmation of mutant phages

The phage plaques obtained were assessed for the presence of mutants by PCR. For this several phage plaques were picked and replicated in *E. coli* BL21 for 2–3 hours. Then phages were recovered by addition of chloroform, followed by centrifugation at 12,000 × *g* for 15 min, and collection of the supernatant. Phage’s DNA was quickly extracted using Proteinase K at 37 °C for 40 min, followed by inactivation at 100 °C for 15 min. PCR reactions were performed using the confirmation primers of Supplementary Table S1 using Kapa Taq DNA polymerase, in reaction volumes of 25 μL, at an annealing temperature of 50 °C. Sizes were confirmed in a 1% SGTB agarose gel using the Low Molecular Weight DNA ladder.

### Phage purification by Cesium Chloride density gradient centrifugation

The mutant phages were purified by cesium chloride density gradient centrifugation. A step gradient of cesium chloride solutions (5.7 mL) was layered on Beckman centrifuge tubes, from the least to the most dense (ρ = 1.33, 1.45, 1.50 and 1.70 g.mL^−1^), adding each new layer to the bottom of the tube. The phage solution (15.2 mL containing 0.5 g of cesium chloride per mL of solution to avoid osmotic shock) was carefully layered over the gradient. The tubes were centrifuged at 28,000 × *g* for 3 h at 4 °C (Beckman Optima Ultracentrifuge XL-80K) in a SW28 rotor. After centrifuging, the band formed by the purified phage was recovered using a syringe with a 20 gauge needle to perforate the side of the centrifuge tubes. Cesium chloride was removed by dialysis against SM buffer at 4 °C.

### Stability of the mutation

The stability of the mutation was assessed over several generations. For this, the T7::PhoE phage was propagated on its host for 4–6 h, followed by centrifugation (6000 × *g*, 10 min, 4 °C) and filtration (0.2 μm) for phage recovery. This procedure was repeated several times, using the phage obtained from the previous propagation. The presence of the mutation was confirmed by PCR, as described above, and confirmed by sequencing at StabVida (Portugal). Each propagation of 4–6 hours was considered as one phage generation, and a total of 10 generations were assessed.

### Transmission electron microscopy

The mutant and wild type-phages were observed by TEM. For this, 15 μL of each purified phage sample were transferred to a 400 mesh copper grid with a pure carbon film (Electron Microscopy Sciences), incubated for three minutes on the grid and removed using filter paper. Grids were then washed with two drops of Milli-Q water and the water removed using filter paper. Then, four assessments were performed: i) no treatment, with samples on grid left to dry without further treatment; ii) negative staining with 1% uranyl acetate solution, performed for 30 seconds followed by removal of all liquid using filter paper; iii) exposure of the dried grids to osmium vapours (from recrystallized osmium tetroxide) for 1.5 h; and iv) incubation with glutaraldehyde/paraformaldehyde fixative for 10 min, washing with two drops of water, drying and exposure for 1.5 hours to osmium vapor. Imaging was performed using a JEOL JEM 1011 transmission electron microscope (JEOL Ltd.).

### High Performance Thin Layer Chromatography

Normalized (same concentration) purified phage isolates were freeze-dried overnight at −52 °C. Total lipid extracts were prepared according to the lipid extraction protocol by Bligh and Dyer^56^. Briefly, the freeze-dried phages were dissolved in 1 mL Milli-Q water, re-suspended in 3.75 mL chloroform-methanol (1:1) and shaken vigorously. Then, 1.25 mL chloroform and 1.25 mL demineralized water were added and the samples vigorous vortexed. Two phases were separated by centrifugation at 1000 × *g* for 5 min. The chloroform phase was collected and washed once with 2.25 mL authentic upper phase. The chloroform phase was again isolated by centrifugation and evaporated under a stream of nitrogen. The lipid pellet was subsequently dissolved in 100 μL methanol and centrifuged briefly to pellet insoluble particles. Five μL total lipid extract were spotted on an activated silica60 HPTLC plate (Merck Millipore, VWR). Chromatograms were developed with a hexane/ethyl acetate (7:3) mobile phase. The dried HPTLC plate was sprayed with a mist of 20% sulfuric acid in ethanol and charred with a heat blower at 160 °C.

### Dynamic light scattering to determine phage charge

DLS was performed to determine possible changes on the surface charge of the mutant phage caused by the incorporation of phospholipids on the capsid. For this, phage stocks were diluted in sterile Milli-Q water to obtain a phage concentration of 10^9^ pfu.mL^−1^. These concentrated phage solutions were used for the measurement of zeta potential in disposable Folded Capillary Cells (Malvern Instruments, DTS1070) using a Zetasizer Nano ZS (Malvern Instruments). Measurements were made in triplicate, in three independent experiments, at a temperature of 25 °C.

### Mutant phage infectivity

To evaluate potential changes in the mutant’s phage infectivity, the one-step growth curve of the wild-type T7 and mutant T7::PhoE was determined as previously described with slight modifications^57^. Briefly, an overnight culture of BL21 was diluted 1:20 in LB medium and grown at 37 °C, 200 rpm until an OD_600_ of 0.35. Bacteria were infected with the wild type or mutant phages at an MOI of 0.001. Samples were taken at short time intervals for a total of 90 min, immediately 10-fold serially diluted in SM buffer, and the phage titre determined using the double agar technique. The latent period, eclipse time and burst size of the phages was determined from the one step growth curve as described by Ellis and Delbrück (1939). The experiments were performed in triplicate and the results presented as mean ± standard deviation.

### Phage survival at different pH values

Phage solutions of wild-type T7 and mutant T7::PhoE were prepared at initial concentrations of 10^8^ pfu.mL^−1^ in 0.2% (wt/vol in water) NaCl adjusted to different pH values (2.5, 3.5, 4.5, and 5.7) using hydrochloric acid, and incubated at different temperatures (38, 40, and 42 °C), with rocking, to simulate conditions encountered in the GIT of animals (see ref.^58^ for more information on these conditions). Samples were taken at different time periods (15, 30, 45, 60, 75, 120, 180, 240, 300 and 1440 min) and immediately 10-fold serially diluted in SM buffer for phage titre determination. The experiments were performed in triplicate.

### Phage capacity to amplify after being subjected to acidic conditions

Phage solutions of wild-type T7 and mutant T7::PhoE were prepared at initial concentrations of 10^8^ pfu.mL^−1^ in 0.2% (wt/vol in water) NaCl adjusted to a pH of 4.5 and incubated at 40 °C with rocking for 3 hours. Then 200 μL of each solution were added to 5 mL of an *E. coli* BL21 culture with an optical density at 600 nm of 0.3, and incubated for 3 h to assess the capacity of the phages to amplify after being subjected to acidic pH, by phage titre determination.

### Reverting phenotype assays with phospholipase

The presence of phospholipids on the capsid of the mutant phages was assessed by enzymatic degradation with an enzyme (phospholipase) that specifically cleaves phospholipids. For this, wild-type and mutant phages, at initial concentrations of 10^8^ pfu.mL^−1^, were subjected to the activity of Phospholipase A2 (Sigma-Aldrich) at a concentration of 10 U/mL, in 0.2% (wt/vol) NaCl (pH of 7). Solutions were incubated for 1 h at 37 °C, added to LB adjusted to a pH of 4 and again incubated at 37 °C. Samples were taken after 0, 60, 180 and 300 min, and immediately 10-fold serially diluted in SM buffer for phage titer determination using the double agar overlay plaque assay as described by Kropinski *et al*.^59^. Control solutions without phospholipase were used. The experiments were performed in triplicate and the results presented as mean log survival ± standard deviation.

### Phage stability in enzymatic fluids

The stability of the wilt type and mutant phages in gastric and pancreatic enzymatic conditions was assessed. For this, simulated gastric fluid (SGF) comprised of 3.2 mg.mL^−1^ pepsin (Amresco) in 0.2% (wt/vol) NaCl at pH 3.5, and artificial pancreatic fluid (APF) comprised of 1 mg.mL^−1^ pancreatin (Biocatalysts) in 0.2% (wt/vol) NaCl at pH 8.0 were prepared. Phages were added to the pre-warmed solutions at 37 °C at a concentration of 10^8^ pfus.mL^−1^ and incubated at 37 °C. Samples were taken at 0, 5, 30, 60, 90 and 120 min and immediately 10-fold serially diluted in SM buffer to determine phage titre. Controls with the same conditions but without the enzymes were used. The experiments were repeated twice and the results presented as mean log survival ± standard deviation of the phages in the enzymatic fluids compared to the respective controls.

### Phage stability in bile salts

The stability of the wild type and mutant phages in bile salts was determined. For this, simulated bile fluid (SBF) comprised of 2% (wt/vol) porcine bile extract (Sigma-Aldrich) was prepared. Phages were added to the pre-warmed solution at 37 °C at a concentration of 10^8^ pfus.mL^−1^ and incubated at 37 °C. Samples were taken at 0, 1 and 3 h and immediately 10-fold serially diluted in SM buffer to determine phage titre. Controls with the same conditions but without the bile salts were used. The experiments were repeated twice and the results presented as mean log survival ± standard deviation of the phages in bile salts compared to the control.

### Statistical analysis

Statistical analysis of the data was performed using the independent samples t-test of the software GraphPad Prism 5, considering a significance level of 95%.

## Additional Information

**How to cite this article**: Nobrega, F. L. *et al*. Genetically manipulated phages with improved pH resistance for oral administration in veterinary medicine. *Sci. Rep.* **6**, 39235; doi: 10.1038/srep39235 (2016).

**Publisher's note:** Springer Nature remains neutral with regard to jurisdictional claims in published maps and institutional affiliations.

## Supplementary Material

Supplementary Information
