# Supplementary material for: Genetically manipulated phages with improved pH resistance for oral administration in veterinary medicine
Source: Sci Rep. 2016 Dec 15;6:39235. doi: 10.1038/srep39235 (PMC5157022; doi:10.1038/srep39235)
Supplement: Supplementary Information [file srep39235-s1.pdf]

Supplementary Information

Genetically manipulated phages with improved pH resistance for oral administration in veterinary medicine

Franklin L. Nobrega[1], Ana Rita Costa[1], José F. Santos[1], Melvin F. Siliakus[2], Jan W. M. van Lent[3], Servé W. M. Kengen[2], Joana Azeredo[1,*], Leon D. Kluskens[1]

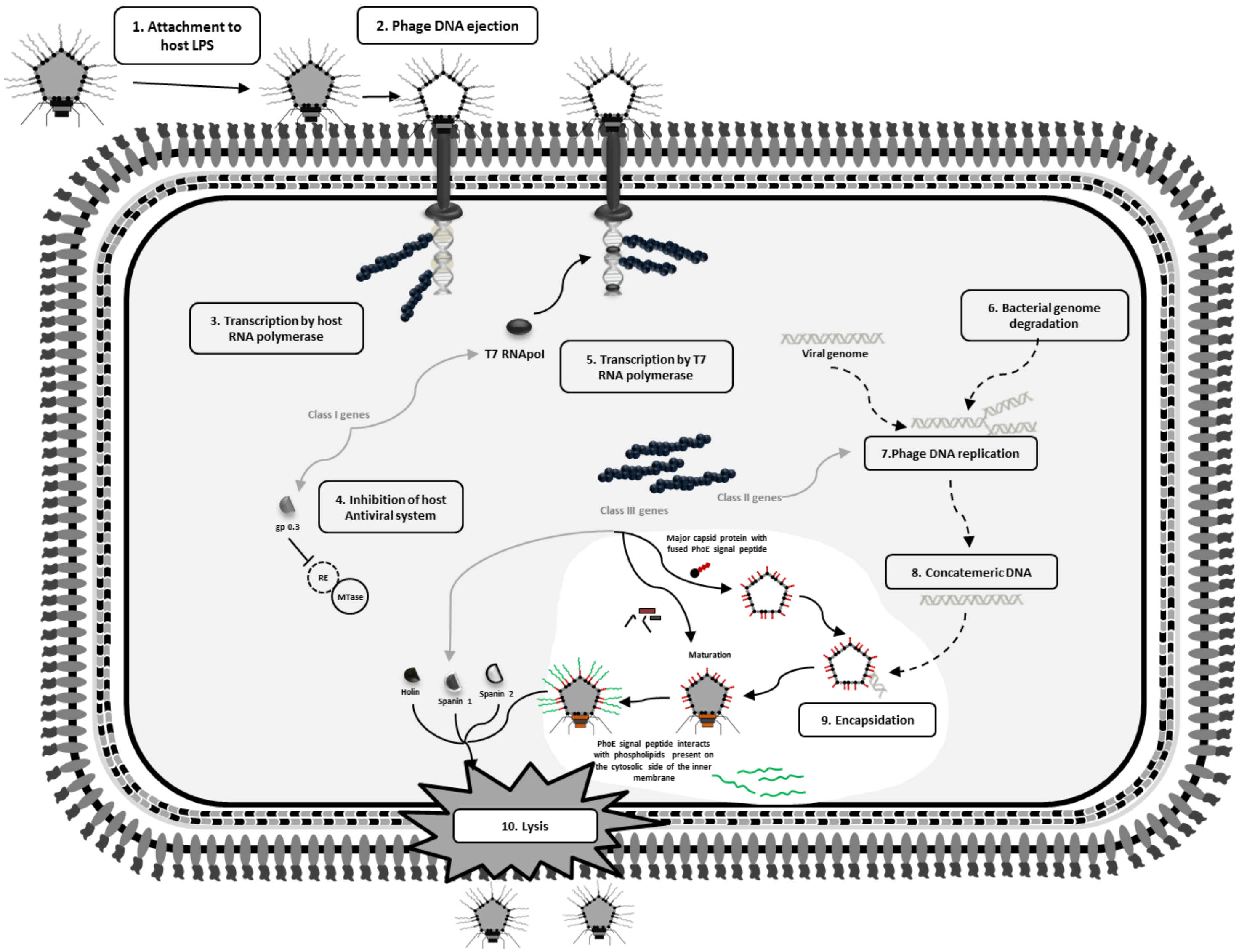

Supplementary Figure S1. Replication of T7::PhoE inside *Escherichia coli*. During replication inside the bacteria, phage T7::PhoE, due to the presence of the PhoE signal peptide on the capsid, signals the incorporation of phospholipids synthesized at the cytoplasmic side of the inner membrane, creating a phospholipid coating.

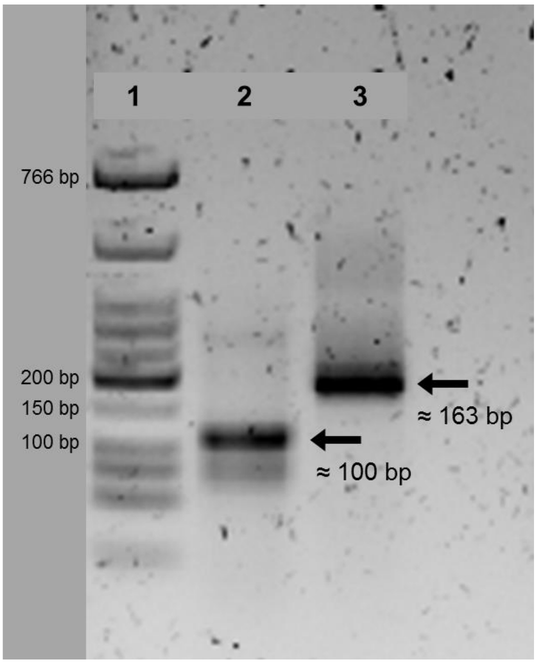

Supplementary Figure S2. PCR confirmation of PhoE signal peptide sequence insertion in phage T7 genome. Lane 1 – Low molecular weight ladder (NEB); Lane 2 – Wild-type T7; and Lane 3 – T7::PhoE. Expected size of approximately 100 bp for wild type T7 and 163 bp for T7::PhoE, run in a 1% SGTB agarose gel.

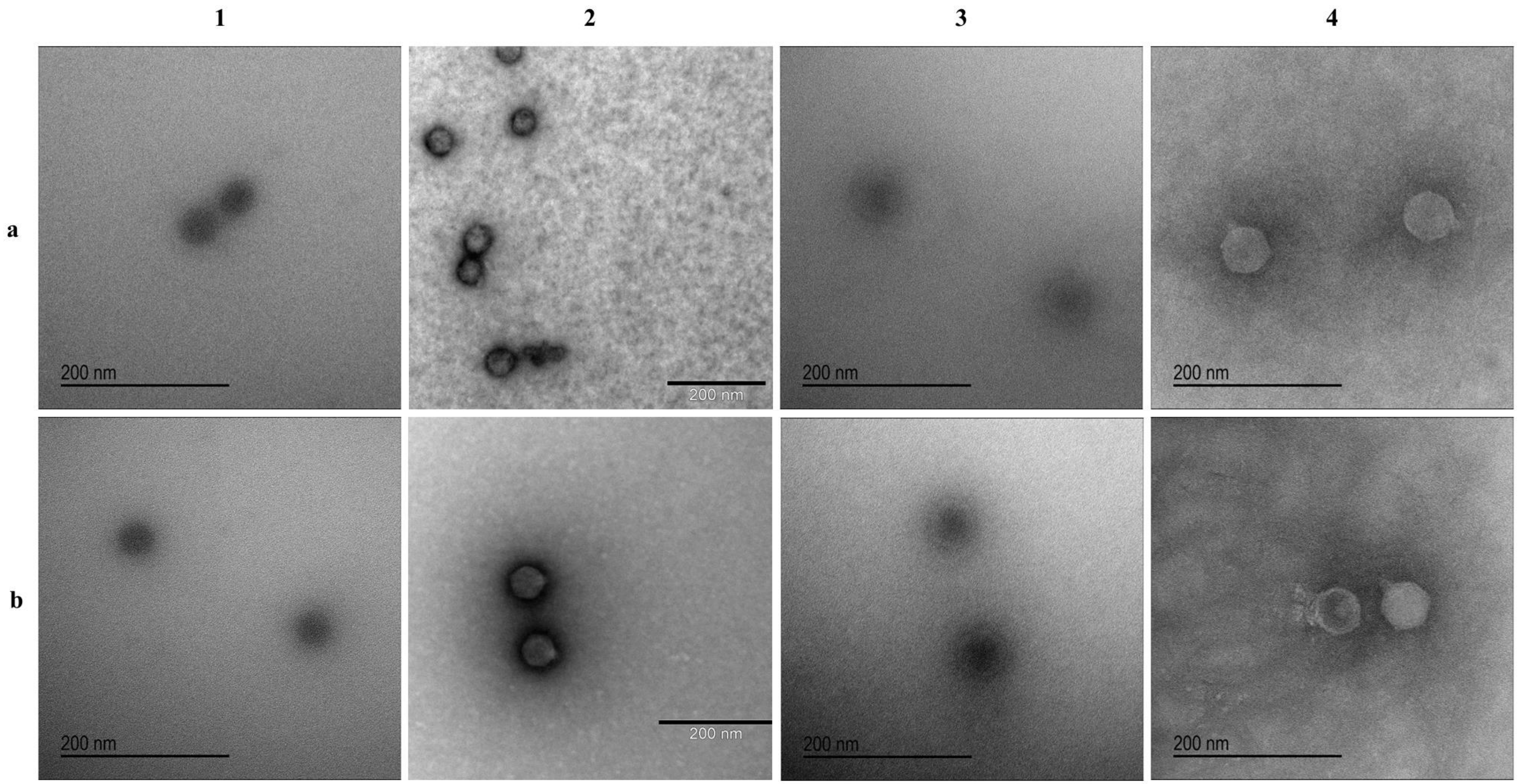

Supplementary Figure S3. Transmission electron microscopy images of the phages. a) wild-type T7 and b) mutant T7::PhoE phages. Images of the phages in a 400 mesh copper grid with a carbon film with 1) no treatment, 2) uranyl acetate negative staining, 3) osmium tetroxide vapours for 1.5h and 4) 10 min glutaraldehyde/paraformaldehyde fixation with with osmium tetroxide vapours for 1.5h.

Supplementary Table S1. Primers used to construct the recombineering substrate for BRED and to confirm the mutation.

PRIMER	SEQUENCE (5' – 3') [a,b]	
PhoE.fw	GTGGTCTTCGCCCAGAAGCTGCTGGTGCAGTGGTTTTCAAAGTGGAGTAAATGAAGAAATCTACGTTGGCACTTGT**CGTGATGGG**	To construct PhoE recombineering substrate for BRED
PhoE.rv	AACACTCGCCTCTTCGGGACTAGCAGCGACCGTTGAGGCCACCCCCAGCAAGCCTGAACAGACGCACTTGCCACGATG**CCCATCACG**	
PhoE.conf.fw	GTGGTCTTCGCCCAGAA	To confirm the presence of the PhoE signal peptide on the mutant phages
PhoE.conf.rv	AACACTCGCCTCTTCGG	

[a] Underlined are the homology regions for the T7 major coat protein

[b] **Bold** is the overlapping region of the primers
